# Functional Expression of Rat Na_v_1.6 Voltage-Gated Sodium Channels in HEK293 Cells: Modulation by the Auxiliary β1 Subunit

**DOI:** 10.1371/journal.pone.0085188

**Published:** 2014-01-03

**Authors:** Bingjun He, David M. Soderlund

**Affiliations:** 1 College of Life Sciences, Nankai University, Tianjin, China; 2 Department of Entomology, Cornell University, Geneva, New York, United States of America; Monell Chemical Senses Center, United States of America

## Abstract

The Na_v_1.6 voltage-gated sodium channel α subunit isoform is abundantly expressed in the adult rat brain. To assess the functional modulation of Na_v_1.6 channels by the auxiliary β1 subunit we expressed the rat Na_v_1.6 sodium channel α subunit by stable transformation in HEK293 cells either alone or in combination with the rat β1 subunit and assessed the properties of the reconstituted channels by recording sodium currents using the whole-cell patch clamp technique. Coexpression with the β1 subunit accelerated the inactivation of sodium currents and shifted the voltage dependence of channel activation and steady-state fast inactivation by approximately 5–7 mV in the direction of depolarization. By contrast the β1 subunit had no effect on the stability of sodium currents following repeated depolarizations at high frequencies. Our results define modulatory effects of the β1 subunit on the properties of rat Na_v_1.6-mediated sodium currents reconstituted in HEK293 cells that differ from effects measured previously in the *Xenopus* oocyte expression system. We also identify differences in the kinetic and gating properties of the rat Na_v_1.6 channel expressed in the absence of the β1 subunit compared to the properties of the orthologous mouse and human channels expressed in this system.

## Introduction

Voltage-gated sodium channels open and close on a millisecond time scale in response to changes in cell membrane potential. This activation/inactivation cycle mediates the transient influx of sodium ions that underlies the electrical action potential in most types of excitable cells [1]. Native sodium channels are thought to exist as heteromultimers comprising one large (∼260 kDa) α subunit and either one or two smaller (33–36 kDa) auxiliary β subunits [2], [3]. The α and β subunits of voltage-gated sodium channels are encoded by multi-gene families. Mammalian genomes contain nine genes for sodium channel α subunit isoforms, designated Na_v_1.1 - Na_v_1.9 [4], [5], and four genes for sodium channel β subunits, designated β1–β4 [3].

Heterologous expression studies in *Xenopus* oocytes and transfected mammalian cells have identified the discrete functional roles of sodium channel α and β subunits. The α subunit forms the ion pore and contains structural domains that confer voltage-dependent gating and the pharmacological properties of the channel [2]. The β subunits modify channel gating, regulate channel expression in the plasma membrane, and contribute to cell adhesion and cell-cell communication [3]. Individual neurons express multiple sodium channel α and β subunit isoforms and contain multiple functionally and pharmacologically distinct sodium channel subunit complexes [6], [7], [8]. However, the subunit compositions of native sodium channel complexes remain to be established.

The Na_v_1.6 isoform is widely expressed in the central and peripheral nervous systems [4] and is the most abundant α subunit isoform in the brains of adult rats [9]. Na_v_1.6 is the predominant isoform at nodes of Ranvier and in regions of brain axons associated with action potential initiation, as well as in presynaptic and postsynaptic membranes of the neocortex and cerebellum [10], [11]. This pattern of expression implies important roles for Na_v_1.6 sodium channels in both electrical and chemical signaling in the brain. A null mutation of the Na_v_1.6 ( = Scn8a) gene in mice, termed “motor endplate disease” (*med*), impairs synaptic transmission at neuromuscular junctions and causes severe paralysis, muscle atrophy and juvenile death [12]. The coincident expression of the Na_v_1.6 and β1 sodium channel subunits in many brain regions [7], [13], [14] suggests that Na_v_1.6 sodium channels may coassemble in heteromultimeric complexes with the β1 subunit *in vivo*. Moreover, the reciprocal interaction between the Na_v_1.6 and β1 subunits to promote neurite outgrowth and determine sodium channel localization [15] identifies a specific functional association between these two subunits.

The only previous studies of the modulatory effects of β subunits on rat Na_v_1.6 sodium channels employed the *Xenopus* oocyte expression system [16], [17]. Whereas the *Xenopus* oocyte system readily permits manipulation of the subunit structure of heteromultimeric channel complexes, the properties of channels in the oocyte membrane environment often differ from the properties of the same channels in native cells, presumably due to differences in membrane structure and post-translational modification [18]. Expression in human embryonic kidney-derived cell lines such as HEK293 offers an alternative system for the functional reconstruction of ion channel complexes that overcomes many of the limitations of the *Xenopus* oocyte system [19]. In light of the importance of the β1 subunit as a modulator of the function and pharmacology of rat Na_v_1.6 sodium channels in the oocyte expression system [16], [17], [20] we undertook the present study to characterize the impact of coexpression with the rat β1 subunit on the functional properties of rat Na_v_1.6 channels expressed in HEK293 cells. Here we describe the functional expression rat Na_v_1.6 sodium channels in HEK293 cells alone or in combination with the rat β1 subunit and compare the properties of the resulting Na_v_1.6 and Na_v_1.6β1 channels. Our results identify modulatory effects of the β1 subunit on the kinetics and gating of Na_v_1.6 sodium channels when expressed in HEK293 cells that differ from its effects on Na_v_1.6 sodium channels expressed in the *Xenopus* oocyte system.

## Materials and Methods

### Sodium Channel Subunit cDNAs

The cloned rat Na_v_1.6 voltage-gated sodium channel α subunit cDNA was provided by L. Sangameswaran (Roche Bioscience, Palo Alto, CA) and the cloned rat sodium channel β1 subunit cDNA was provided by W.A. Catterall (University of Washington, Seattle, WA). Each cDNA insert was subcloned into the vector pcDNA3.1 (Invitrogen, Carlsbad, CA) and the integrity of each clone was confirmed by DNA sequencing.

### HEK-Na_v_1.6 Cell Lines

HEK293 cells (CRL-1573, lot number 7681666) were obtained from the American Type Culture Collection (ATCC, Manassas, VA) and cultured at 37°C in Dulbecco’s modified Eagle’s medium (DMEM) supplemented with 10% fetal bovine serum (FBS) and 1% penicillin/streptomycin (all from ATCC) in a humidified atmosphere of 5% CO_2_/95% air. Upon receipt cells were passaged twice and then frozen in DMEM+FBS with 5% dimethyl sulfoxide (DMSO) for future use; these stocks were considered to be at “laboratory passage one.” One day before transfection, cells (passage five, 0.5×10^5^ cells/100 µl growth medium without antibiotics) were transferred to a well of a 96-well plate and grown until ∼80% confluent. Cells were transfected using Lipofectamine™2000 (Invitrogen) according to the manufacturer’s protocol and either 254 ng of the Na_v_1.6 plasmid or 200 ng of a mixture of the Na_v_1.6 and β1 plasmids (1∶2 molar ratio). Cells were diluted 1∶10 into 6-well plates 24 h after transfection, incubated in culture medium for an additional 24 h, and then selected for 15 days with culture medium containing G418 (Invitrogen; 800 µg/ml). Clonal colonies (derived from a single cell; ∼50 cells/colony) of G418-selected cells were isolated using cloning rings (Sigma-Aldrich, St. Louis, MO) and maintained in continuous culture under G418 selection (400 µg/ml) for electrophysiological characterization. Clonal cell lines giving cells with whole-cell peak transient sodium currents amplitudes ≥2000 pA were saved as frozen stocks for further use.

### Analysis of Sodium Channel Subunit Expression

First-strand cDNA from transfected sodium current-positive cell lines, synthesized using the SuperScript™ III CellsDirect cDNA synthesis system (Invitrogen), was employed as the template in polymerase chain reaction (PCR) amplifications using pairs of oligonucleotide primers specific for the rat Na_v_1.6 α subunit and the rat β1 subunits as described previously [21].

### Electrophysiology

On the day prior to assay, cells were plated at low density in 35-mm Petri dishes. For electrophysiological assays, cells (24–48 h after plating) were rinsed three times with extracellular perfusion medium that contained (mM): NaCl (140), KCl (5), CaCl_2_ (2), MgCl_2_ (1), and HEPES (10) at pH 7.40 (adjusted with 2 M NaOH). Whole-cell patch clamp recordings were conducted at room temperature (23–27°C) using an Axopatch 200B amplifier (Molecular Devices, Foster City, CA). Cells were perfused at ∼350 µl/min with extracellular medium using a custom-fabricated passive perfusion manifold and a disposable plastic recording chamber insert (∼240 µl volume; Warner Instruments, Hamden, CT). A stock solution of tetrodotoxin (TTX; Sigma Chemical Co., St. Louis, MO) was diluted to a final concentration of 0.5 µM in extracellular medium and applied through the perfusion system. The intracellular solution contained (in mM): NaCl (35), CsF (105), MgCl_2_ (2), EGTA (10), and HEPES (10) at pH 7.20 (adjusted with 2 M CsOH). The final osmolarity of both solutions was 295–305 mOsm. Fire-polished patch electrodes were fabricated from borosilicate glass capillaries (1.5 mm O.D.; 1.0 mm I.D.; World Precision Instruments Inc., Sarasota, FL) using a P-87 puller (Sutter Instruments, Novato, CA) to give a resistance of 1–2 MΩ when filled with intracellular solution. The ground electrode was a bridge of 1% agar in extracellular medium in a glass pipet. Output signals were filtered at 2 kHz and sampled at 50 kHz (DigiData 1322A; Molecular Devices). Voltage errors were minimized using 70–80% series resistance compensation. Leak currents were corrected using the P/4 method [22]. Data were acquired using pClamp 10.2 (Molecular Devices) software. Following the establishment of a stable holding potential (−120 mV) under voltage clamp and measurement of cell capacitance, sodium currents were sampled using 40-ms step depolarizations to −15 mV at a frequency of 0.05 Hz for ∼20 min to achieve stable sodium current amplitudes prior to initiating other protocols. To determine the voltage dependence of activation, cells were clamped at a membrane potential of −120 mV and currents were measured during 40-ms depolarizing test pulses to potentials from −80 mV to 65 mV in 5-mV increments. Persistent currents, operationally defined as the residual current remaining at the end of a 40-ms depolarizing test pulse, were measured at test potentials giving maximal peak transient sodium current in each cell and normalized to the amplitude of the peak current in the same depolarization [23]. Efforts to detect and characterize resurgent currents involved 20-ms step depolarizations from a holding potential of −120 mV to a test potential of 30 mV followed by partial repolarizations for 100 ms to potentials from 20 mV to −80 mV in 5-mV increments. To determine the voltage dependence of steady-state fast inactivation, cells were clamped at a membrane potential of −120 mV followed by a 100-ms conditioning prepulse to potentials from −120 mV to 0 mV in 5-mV increments and then a 40-ms test pulse to −15 mV. For determinations of use dependence, cells were given trains of up to 100 5-ms conditioning prepulses from −120 mV to 10 mV at 20 or 66.7 Hz followed by a 40-ms test pulse from −120 mV to −15 mV.

### Data Analysis

Data were acquired and analyzed using pClamp 10.2 (Molecular Devices) and Origin 8.1 (OriginLab Corp., Northampton, MA). For each cell, currents from activation experiments were converted to sodium conductances and plotted as a function of test potential using the Boltzmann equation [y = (A_1_– A_2_)/(1+e^(x–x0)/dx^)+A_2_] to give values for V_0.5_ (potential causing half-maximal activation) and K (slope factor). Similarly, currents from steady-state inactivation experiments with each cell were plotted as a function of prepulse potential and fitted to the Boltzmann equation. Statistically significant effects of the β1 subunit were identified using Student’s unpaired t-test by analysis in Prism 5.0 (GraphPad Software, La Jolla, CA).

## Results

We obtained clonal cell lines that were stably transformed to express the rat Na_v_1.6 sodium channel α subunit isoform either alone (HEK-Na_v_1.6) or in combination with the rat β1 subunit (HEK-Na_v_1.6β1). We confirmed the translational expression of the desired sodium channel subunits in each cell line by RT-PCR (data not shown). Table 1 summarizes the mean sodium current amplitudes and densities for each cell line. The amplitudes of peak transient sodium currents varied from 1 nA to 20 nA between individual cells in each cell line, with the majority of cells expressing currents in the 2–5 nA range.

**Table 1:** Expression of sodium currents in HEK293 cells transfected with the Na_v_1.6 sodium channel α subunit alone or cotransfected with the β1 subunit.

Cell line	Membrane capacitance (pF)	Peak current amplitude (nA)	Current density (pA/pF)	*n*
HEK-Na_v_1.6	20.9±0.4	5.1±0.4	240±19	57
HEK-Na_v_1.6β1	21.3±0.5	3.0±0.2	139±7	51

**Figure 1:**
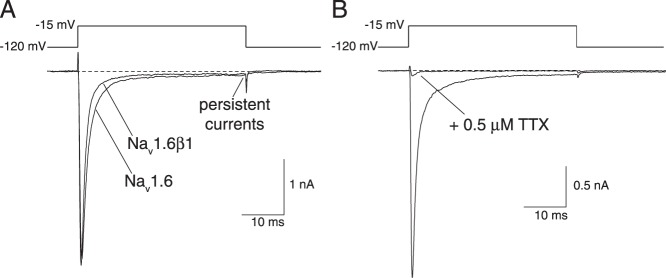
Sodium currents recorded from HEK-Na_v_1.6 and HEK-Na_v_1.6β1 cells. (A) Representative sodium current traces recorded from HEK-Na_v_1.6 and HEK-Na_v_1.6β1 cells following 40-ms depolarizations from −120 mV to −15 mV. (B) Sodium currents recorded from a HEK-Na_v_1.6β1 cell before and after exposure to 0.5 µM TTX. Dashed lines indicate zero current.

Fig. 1A shows representative sodium current traces recorded from HEK-Na_v_1.6 and HEK-Na_v_1.6β1 cells. Table 2 summarizes the kinetic parameters of sodium currents, such as those illustrated in Fig. 1A, obtained in recordings from multiple cells derived from each cell line. We used the time interval from membrane depolarization to the peak current as an indirect index of the rate of channel activation. Coexpression of the Na_v_1.6 α subunit with the β1 subunit had no effect on the time to peak current. We obtained inactivation time constants (τ_inact_) for currents in each cell line by fitting the falling phase of peak transient currents to a first-order decay model. Coexpression of Na_v_1.6 with the β1 subunit significantly accelerated the decay of the peak current.

**Table 2:** Effects of coexpression with the β1 subunit on the kinetics of sodium current activation and inactivation and amplitudes of persistent currents in HEK293 cells expressing Na_v_1.6 sodium channels.

	Peak current activation and inactivation^a^			Persistent current^b^	
Channel	time to peak	τ_inact_	*n*	percent of peak	*n*
Na_v_1.6	1.00±0.03	1.38±0.05	63	1.48±0.17	64
Na_v_1.6β1	0.99±0.02	1.20±0.03^c^	60	1.45±0.10	65

^a^ Time to peak current (ms) and first-order time constant (τ_inact_, ms) of sodium current inactivation; values are means ± SE for the indicated number of replicate experiments with different cells.

^b^ Current measured at the end of a 40-ms depolarizing pulse expressed as a percentage of the peak current in the same depolarization; values are means ± SE for the indicated number of replicate experiments with different cells.

^c^ Significantly different from the value for Na_v_1.6 channels (*P*<0.005).

Previous studies of human [23], [24] and mouse [25] Na_v_1.6 sodium channels expressed in mammalian cell lines in the absence of auxiliary β subunits identified a significant persistent component of the sodium current (≥10% of the peak current) that did not inactivate upon prolonged depolarization. In HEK293 cells expressing the rat Na_v_1.6 channel alone, the persistent component of current (measured at the end of a 40-ms depolarizing pulse as in Fig. 1A) was small (∼1.5% of the peak transient current; Table 2). Coexpression of the Na_v_1.6 α subunit with the β1 subunit had no effect on the amplitude of the persistent current.

Figure 1B shows the effect of TTX (0.5 µM) on a representative sodium current recorded from HEK-Na_v_1.6β1 cells. In this cell, which is typical of all cells in which the action of TTX was examined, TTX blocked the persistent current but did not completely block the peak current. The small residual peak currents measured in this and other cells in the presence of 0.5 µM TTX correspond to the TTX-resistant component of the endogenous multicomponent voltage-gated cation current described previously in the parental HEK293 cell line [26].

**Figure 2:**
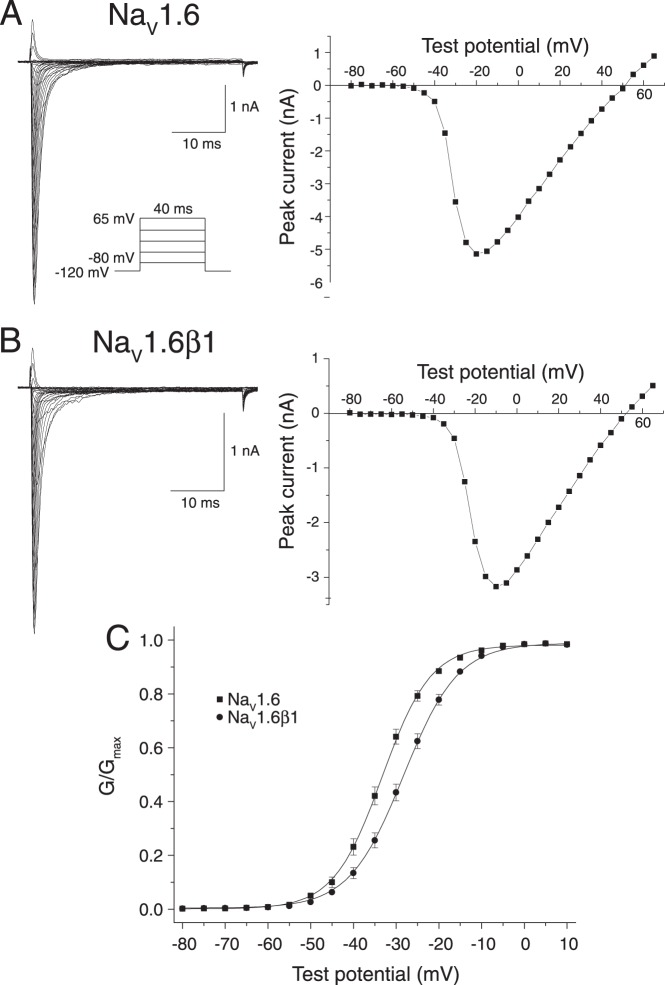
Voltage-dependent activation of Na_v_1.6 and Na_v_1.6β1 sodium channels expressed in HEK293 cells. (A) Representative current traces recorded from a HEK-Na_v_1.6 cell using the indicated pulse protocol (left) and the plot of peak sodium current in these traces as a function of test potential (right). (B) Representative current traces recorded from a HEK-Na_v_1.6β1 cell using the pulse protocol shown in Panel A and the plot of peak sodium current in these traces as a function of test potential. (C) Conductance – voltage plots for the activation of Na_v_1.6 and Na_v_1.6β1 channels. Peak sodium currents such as those in Panels A and B were transformed to conductances (G) using the equation G = I/(V_t_–V_rev_), where I is the peak current, V_rev_ is the reversal potential, and V_t_ is the voltage of the test potential; conductances were then normalized to the maximum conductance (G_max_) for that cell. Values are means of 64 (Na_v_1.6) or 65 (Na_v_1.6β1) separate experiments with different cells; bars show SE values larger than the data point symbols. Curves were fitted to the mean values using the Boltzmann equation.

Figure 2 describes the effects of the β1 subunit on the voltage-dependent activation of Na_v_1.6 sodium channels expressed in HEK293 cells. Figure 2A shows families of sodium currents, recorded using the indicated pulse protocol, from an individual representative cell expressing rat Na_v_1.6 sodium channels and current – voltage plot of the peak transient sodium currents in these traces. Figure 2B shows sodium current traces and current – voltage relationships from an individual representative cell expressing Na_v_1.6β1 sodium channels. Figure 2C shows conductance – voltage plots of data obtained from multiple cells expressing rat Na_v_1.6 sodium channels either in the absence or presence of the β1 subunit, and Table 3 summarizes the statistical analysis of these data. The β1 subunit caused a statistically-significant 6.6-mV depolarizing shift in the midpoint potential (V_0.5_) for channel activation that was accompanied by a significant increase in the slope factor (K) of the activation curve.

**Table 3:** Effects of coexpression with the β1 subunit on the voltage dependence of activation and steady-state inactivation of Na_v_1.6 sodium channels expressed in HEK293 cells.^a^

	Activation			Inactivation		
Channel	V_0.5_	K	*n*	V_0.5_	K	*n*
Na_v_1.6	−35.2±0.8	4.19±0.17	64	−68.7±0.7	6.50±0.10	63
Na_v_1.6β1	−28.6±0.8**^b^	4.90±0.12**	65	−63.5±0.8*	6.11±0.08**	66

^a^ Values are means ± SE calculated from fits of the data from the indicated number of individual cells to the Boltzmann equation; V_0.5_, midpoint potential (mV) for voltage-dependent activation or inactivation; K, slope factor.

^b^ Significantly different from the value for Na_v_1.6 channels (*P<0.001; **P<0.0005).

**Figure 3:**
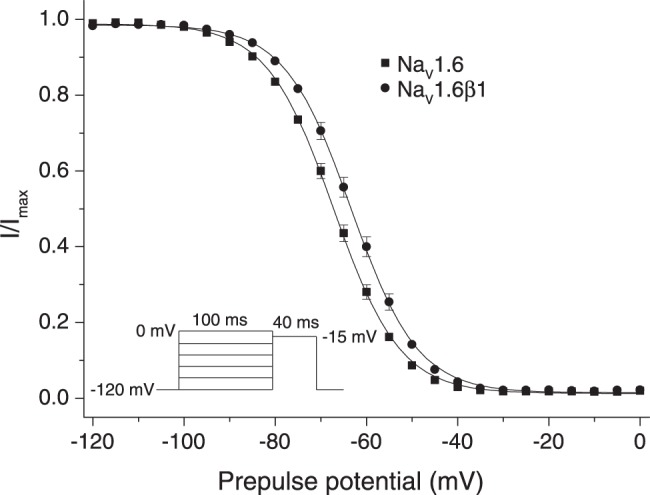
Voltage-dependent steady-state fast inactivation of Na_v_1.6 and Na_v_1.6β1 sodium channels expressed in HEK293 cells. Amplitudes of peak transient currents obtained using the indicated pulse protocol are plotted as a function of prepulse potential. Values are means of 63 (Na_v_1.6) or 66 (Na_v_1.6β1) separate experiments with different cells; bars show SE values larger than the data point symbols. Curves were fitted to the mean values using the Boltzmann equation.

Figure 3 shows the effects of the β1 subunit on the voltage dependence of steady-state fast inactivation of Na_v_1.6 sodium channels expressed in HEK293 cells, and Table 3 summarizes the statistical analysis of these data. The β1 subunit caused a statistically-significant 5.2-mV depolarizing shift in the midpoint potential (V_0.5_) for steady-state inactivation that was accompanied by a significant decrease in the slope factor (K) of the activation curve.

**Figure 4:**
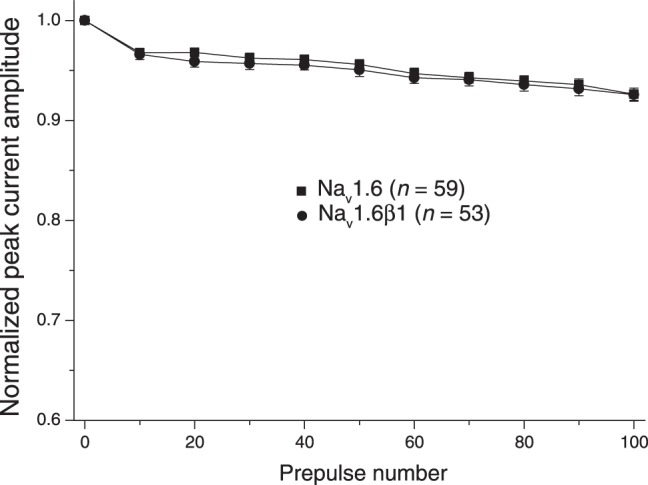
Effect of repeated depolarization on the stability of sodium currents recorded from HEK293 cells expressing Na_v_1.6 and Na_v_1.6β1 sodium channels. Sodium currents were recorded during a 40-ms step depolarization from −120 mV to −15 mV following 0–100 conditioning prepulses (5-ms pulses from −120 mV to 10 mV at 20 Hz). Currents for each cell were normalized to the amplitude of the peak current obtained prior to repeated depolarization. Values are means of the indicated number of separate experiments with different cells; bars show SE values larger than the data point symbols.

Evidence implicating Na_v_1.6 sodium channels in high-frequency firing and the production of resurgent currents in Purkinje neurons [27] led us to assess the impact of coexpression with β1 subunit on these properties in HEK-293 cells expressing rat Na_v_1.6 sodium channels. Figure 4 shows the effect of the β1 subunit on the stability of peak transient sodium currents following high-frequency stimulation. We applied 0–100 brief (5-ms) depolarizing pulses to 10 mV prior to a standard 40-ms test depolarization to −15 mV. Currents carried by Na_v_1.6 sodium channels in the absence of β subunits declined rapidly to ∼95% of the control current within the first 10 prepulses and then stabilized. Coexpression of Na_v_1.6 with the β1 subunit did not affect the stability of the peak current measured using this protocol. We obtained similar results using a prepulse frequency of 66.7 Hz (data not shown). We also employed a conventional pulse protocol, involving partial membrane repolarization following a depolarizing pulse [28], in an effort to identify and characterize resurgent currents in these cells. However, we found no evidence for resurgent currents in either the absence or presence of the β1 subunit.

## Discussion

HEK293 cells and cell lines derived from them are employed extensively as platforms for the transient or stable heterologous expression of neuroreceptor and ion channel proteins [19]. HEK293 cells were derived from human embryonic kidney cells by transformation with sheared adenovirus type 5 DNA [29]. Despite their origin HEK293 cells exhibit some characteristics of neurons, expressing more than 60 neuronal genes such as neurofilament proteins and neuroreceptor and ion channel subunits [19], [30], including low levels of the sodium channel β1A auxiliary subunit [31]. Moreover, our previous electrophysiological studies confirmed the existence of small endogenous voltage-gated cation currents in HEK293 cells. The TTX-sensitive component of the endogenous current, a presumptive sodium current, was associated primarily with the expression of the human Na_v_1.7 sodium channel isoform [26].

We overexpressed the rat Na_v_1.6 α subunit and the rat β1 subunit in HEK293 cells by transformation with the corresponding plasmids and selecting stably-transformed clonal cell lines that exhibited voltage-gated sodium currents under whole-cell patch clamp conditions with amplitudes >1 nA. Each of the cell lines described here exhibited sodium current densities more than tenfold greater than the density of the endogenous multicomponent cation currents in the parental HEK293 cell line [26]. Currents measured in the presence of 0.5 µM TTX (as in Fig. 1B) represent the TTX-resistant component of this current, which constitutes ∼50% of the total endogenous current. The relative amplitudes of peak transient currents measured in the absence and presence of TTX therefore provide a direct assessment of the contamination of the total current by elements of the endogenous cation current. The high level of current expression in HEK-Na_v_1.6 and HEK-Na_v_1.6β1 cells ensured that the whole-cell sodium currents described here were carried predominantly by heterologously-expressed rat Na_v_1.6 sodium channels rather than by endogenous channels. Previous studies showed that the low level of expression of the endogenous human β1A auxiliary subunit in HEK293 cells is insufficient to affect the properties of overexpressed heterologous sodium channel α subunits [32]. Thus, we attribute the sodium currents described here to the heterologously-expressed rat sodium channel subunits or subunit complexes rather than to endogenous channel complexes present in the parental cell line.

This study is the first description of the properties of rat Na_v_1.6 sodium channels in a mammalian cell expression system and the modulation of these properties by the rat sodium channel β1 auxiliary subunit. It is also the first description of the modulation of any Na_v_1.6 ortholog by the β1 subunit of the same species. Coexpression with the rat β1 subunit accelerated the inactivation of peak transient sodium currents carried by Na_v_1.6 channels in HEK293 cells and shifted the voltage dependence of both channel activation and steady-state inactivation in the direction of depolarization. By contrast, we found no effect of the β1 subunit on the latency of the peak current (an indirect measure of rate of activation) or the stability of peak sodium currents following repeated, high-frequency stimulation.

The effects of the rat β1 subunit on the rat Na_v_1.6 sodium channel reported here differ from its effects on the human Na_v_1.6 ortholog in the HEK293 cell expression system [33]. Contrary to our findings, the rat β1 subunit had no effect on the voltage dependence of either activation or steady-state inactivation of the human Na_v_1.6 channel. This result suggests that the rat β1 subunit is not able to interact in an equivalent manner with the rat and human Na_v_1.6 orthologs to modulate voltage-dependent gating and underscores the importance of employing subunits from the same species in the reconstitution of channel function *in vitro*.

Comparison of the results of the present study with those for rat Na_v_1.6 sodium channels expressed in the *Xenopus* oocyte system [17] reveals substantial differences in voltage-dependent gating between expression systems. In the absence of auxiliary β subunits the midpoint potentials for the voltage dependence of both activation and steady-state inactivation of rat Na_v_1.6 sodium channels in HEK293 cells were shifted by 17–22 mV in the direction of hyperpolarization when compared to channels expressed in oocytes. Moreover, in HEK293 cells coexpression with the β1 subunit produced depolarizing shifts in the voltage dependence of activation and steady-state inactivation, whereas in oocytes the β1 subunit had no effect on the voltage dependent gating of rat Na_v_1.6 channels. Coexpression of Na_v_1.6 with the β1 subunit in oocytes enhanced the persistent component of the sodium current, whereas in HEK293 cells the β1 subunit had no effect on persistent current. In both expression systems, however, the β1 subunit accelerated the rate of sodium current inactivation.

Comparison of our results using the HEK-Na_v_1.6β1 cell line with the results of our previous study using the corresponding HEK-Na_v_1.6β1β2 cell line [21] permits us to infer the distinctive contributions of the rat β2 subunit to the properties of heterotrimeric Na_v_1.6β1β2 sodium channel complexes. Such comparisons reveal no discernable effect of the β2 subunit on the kinetics of peak current decay or the voltage dependence of channel activation and steady-state fast inactivation. Thus, we conclude that the β1 subunit is the principal modulator of the properties of Na_v_1.6β1β2 sodium channel complexes based on aspects of channel function that have been examined to date. However, this does not preclude important effects of the β2 subunit in channel trafficking or other channel properties, such as slow inactivation, that remain to be investigated.

The modulatory effects of the β1 subunit on sodium channel function are neither consistent nor predictable across sodium channel α subunit isoforms from the same species. The effects of the rat β1 subunit on the voltage dependence of various rat sodium channel subunit isoforms expressed in HEK293-based cell systems exemplify these isoform-specific effects. Consistent with our results using the rat Na_v_1.6 ortholog, coexpression with the rat β1 subunit shifted the voltage dependence of activation and inactivation of rat Na_v_1.2 channels in the direction of depolarization [34]. By contrast, coexpression with the rat β1 subunit did not significantly affect the voltage dependence of activation and inactivation of rat Na_v_1.3 channels [35] or rat Na_v_1.4 channels expressed in HEK293 cells [36].

Finally, comparison of our data for the rat Nav1.6 channel expressed in the absence of β subunits with published data for the expression the human Na_v_1.6 channel expressed in HEK293 cells [23], [24], [33] and the mouse Na_v_1.6 channel expressed in tsA-201 cells (a subclone of HEK293 cells) [25], reveals apparent species differences in both the persistent component of the sodium current and the voltage dependence of channel gating. Both human and mouse Na_v_1.6 channels gave prominent persistent currents that did not inactivate during a typical depolarizing pulse. The amplitudes of these persistent currents, expressed as a percentage of the peak transient current in the same depolarization, were approximately 12% for mouse channels expressed in TsA-201 cells [25] and up to 26% for human channels expressed by transient transfection in HEK293 cells [23]. By contrast the persistent current in our assays with rat Na_v_1.6 channels was barely detectable at approximately 1.5% of the peak transient current, a value similar to that reported previously for rat Na_v_1.2 channels expressed in TsA-201 cells [25] but much lower than those found in equivalent assays with the orthologous mouse and human channels.

The rat Na_v_1.6 channel, similar to the human ortholog, also differed from the mouse ortholog in its voltage dependence of activation and steady-state fast inactivation. Table 4 summarizes available data for the voltage dependent gating of rat, human and mouse Na_v_1.6 channels expressed in human embryonic kidney-derived cell lines in the absence of β subunits. Rat Na_v_1.6 channels gave midpoint potentials for activation and steady-state fast inactivation that were similar to or somewhat more than more negative than those measured in two separate studies with the orthologous human Na_v_1.6 channels [23], [33] but approximately 21 mV more negative than those measured for the orthologous mouse Na_v_1.6 channels [25]. The relatively depolarized activation gating of the mouse Na_v_1.6 ortholog is confirmed by expression studies of mouse Na_v_1.6_R_ (mutated to confer resistance to TTX) in ND7/23 cells, a dorsal root ganglion-derived cell line [37] in which sodium currents carried by the heterologously-expressed Na_v_1.6_R_ channels are isolated from endogenous sodium currents by recording in the presence of TTX. These results suggest that intrinsic species differences in the gating properties of orthologous Na_v_1.6 channels may exist despite their high amino acid sequence identity. Differences in channel structure capable of conferring these differences remain to be determined.

**Table 4:** Comparison of the voltage dependence of activation and steady-state fast inactivation of rat, human and mouse Na_v_1.6 sodium channels expressed in human embryonic kidney-derived cell lines in the absence of auxiliary β subunits.

		V_0,5_, mV	
Species	Host cell	Activation	Inactivation
Rat^a^	HEK293	−35.2	−68.7
Human	HEK293	−29.2^b^, −36.7^c^	−53.4^b^, −74.3^c^
Mouse^d^	TsA-201	−13.6	−47.4

^a^ Data from this study.

^b^ Data from Burbidge *et al.* [23].

^c^ Data from Zhao *et al.* [33].

^d^ Data from Chen *et al.* [25].
